# Modified natural cycle frozen-thawed embryo transfer in patients with repeated implantation failure: An observational study

**Published:** 2016-07

**Authors:** Soheila Arefi, Ahmad Hoseini, Fattaneh Farifteh, Hojjat Zeraati

**Affiliations:** 1 *Avicenna Research Institute, Shahid Beheshti University, Tehran, Iran.*; 2 *GIVAR (Genetic & In Vitro Assisted Reproduction) Center, Erfan hospital, Tehran, Iran.*; 3 *Cellular and Molecular Biology Research Center, Shahid Beheshti University, Tehran, Iran.*; 4 *Tehran University of Medical Sciences, Tehran, Iran.*

**Keywords:** *Embryo transfer*, *Hormone replacement therapy*, *Embryo implantation*

## Abstract

**Background:**

Natural endometrium in Frozen-thawed Embryo Transfer (FET) may have some benefits upon implantation in patients with Repeated Implantation Failure (RIF). It might be due to possible differences between natural and stimulated endometrial growth factors and cytokins secretions.

**Objective:**

The objective of this study was to compare the pregnancy rate of FET on modified natural cycle versus hormone replacement therapy (HRT) cycle endometrium in patients with RIF.

**Materials and Methods:**

In this observational study the pregnancy rate of patients with RIF undergoing day 3 FET in natural cycle endometrium (group 1, n=56), were compared with another group of patients with RIF in whom frozen-thawed day 3 embryos were transferred on HRT cycle (group 2, n=52).

**Results:**

The pregnancy rate in group 1 was 41.07%, compared with the pregnancy rate of group 2; 36.5% (p=0.63). The abortion rate was not significantly different among the groups.

**Conclusion:**

It can be concluded that FET in a modified natural cycle is comparable with HRT cycle in patients with RIF.

## Introduction

The reasons for repeated In-vitro Fertilization (IVF) failure may be defective endometrial receptivity, embryonic chromosomal abnormality, or multiple factorials. Embryonic defect may be due to sperm and/or oocyte genetic abnormality, as well as meiotically or post-zygoticall chromosomal abnormality of early human embryos (1-3). 

Defective endometrial receptivity may be due to molecular dysregulation or morphological disruption. It has been shown that inherited thrombophilia such as factor V Leiden (FVL) mutation, prothrombin mutation, methylene tetra hydrofolate reductase (MTHFR) mutation, and deficiencies in proteins S and C and antithrombin III, and acquired thrombophilia like lupus anticoagulant and anticardiolipin may have critical role in implantation failure (4, 5). Elevation of uterine natural killer (NK) cells, altered expression of LIF (leukaemia inhibitory factor), and IL-15 (interleukin 15), IL-12,IL-15 high IL-1β and low interferon-γ and IL-10 are considered as molecular reasons for implantation failure (6-9). 

Intrauterine abnormalities like hyperplasia, polyp, myoma, and adhesions have been proposed as the possible causes of impaired implantation. There is no doubt that hysteroscopy should be performed in cases with RIF when there is suspicion of intrauterine pathology (10). Morphologically poor endometrium is a big obstacle in reproductive success due to difficulty of the non-responsive endometrium treatment. This has been shown in many studies, various empirical treatments like pentoxifylline and tocopherol, and Silfendanil may improve the endometrial response after priming by estrogen, as measured by ultrasonographic thickness (11, 12). 

Natural endometrium frozen-thawed embryos (FET) may have some benefits on implantation in patients with Repeated Implantation Failure (RIF) and even RIF with poor endometrium in whom never get pregnant after transferring good quality embryos. Analysis of proteins in endometrial fluid immediately before embryo transfer (ET) provides important information about endometrial receptivity. It has been shown that the concentration of cytokines and growth factors are different between natural and stimulated endometrial secretion (13). 

In this study we assessed the pregnancy rate in FET cycles in a natural compared to hormone treated endometrium, in patients with the history of RIF, with and without a history of poor endometrium.

## Materials and methods

This retrospective study has been performed in Erfan Hospital infertility clinic from April 2009 to April 2010. The patients were 20-38 years old women underwent ICSI. The proposal was approved by the ethical committee of Erfan hospital. 

All patients were stimulated with standard long protocol, gonadotrophin releasing hormone (GnRH) agonist (Superfact, Aventis Pharma, Germany) from day 21 of the cycle proceeding the stimulation cycle. Then from the second to the third day of the stimulation cycle, patients were received FSH (Gonal-F, Merck Serono, Germany) 150-300 units daily. When at least three follicles had a diameter >18 mm, 10,000 IU unit of HCG was administered. After 34-36 hrs, puncture of ovaries was done. 108 patients with more than two RIFs who were candidates for FET, were included in the study. 

All extra embryos were cryopreserved on day three by vitrification. Flowcytometry, autoantibodies profile like anti-Cardiolipin (aCL), Lupus Anticoagulant (LA), anti-Phosphatidylserine (aPS), anti-Phosphatidylethanolamine (aPE), and Antinuclear Antibodies (ANA), Anti DNA, serum homocystein, karyotype and hysteroscopy were performed in all women with the history of implantation failure. 

The exclusion criteria were intrauterine pathology, Asherman syndrome, thrombophilia, immunological and genetic problems, oligo/un-ovulation, and severe male factor infertility. All patients signed informed consent before the treatment. The outcomes after FET, on natural endometrium (group 1, n=56) were compared with the outcome of FET on HRT cycle, (group 2, n=52) in patients with RIF. The policy to transfer up to 3 embryos in patients with RIF was adopted. All frozen-thawed embryos were transferred on day three in all groups. Cyclogest (Actavis, UK) 400 mg twice daily were given to all patients as luteal phase support. The main outcome measured was pregnancy rate per ET procedure. Secondary outcomes were abortion rate and multifetal pregnancy. 

**Modified Natural cycle protocol**

Serial monitoring of ovulation was done by regular ultrasound evaluation and serum LH in the 1^st^, 7^th^, and 10^th^-11^th^ day of the cycle in group 1 and group 2. When the LH surge was confirmed the serum LH monitored the day after. Ovulation was induced using 10,000 IU of urinary hCG (Choriomon, IBSA, Switzerland) when the dominant follicle diameter was >16 mm and endometrial thickness >7 mm, detected by ultrasound. HRT prepared endometrium protocol: HRT was started Estradiol valerate 2 mg/day, and increased by 2 mg every 3 days in previously down regulated cycle. Cyclogest 400 mg daily was given for three days before ET, after reaching at least ≥7 mm triple line endometrium. 

**Cycle outcome**

Pregnancy was defined as a positive serum β-hCG test 14 days after ET. Clinical pregnancy was defined by the presence of gestational sac with positive fetal heartbeat on ultrasonography, approximately 2-3 wks after a positive pregnancy test.

**Statistical analysis**

The Mean±SD were determined for each study group. Data were analyzed by ^2^ and Fisher exact test, independent t-test multiple comparison procedure to calculate the significance. P<0.05 value between study groups was taken as statistically significant.

## Results

There were no significant differences in age, duration of infertility, number of previous attempts, and number of transferred embryos between groups (Table I). The natural cycle had a higher trend of clinical pregnancy rate without reaching statistical significance in patients with repeated IVF failure and normal endometrium in whom embryos were transferred in natural endometrium (41.07, 23 out of 56), in comparison with the HRT cycle patients (36.5%) (19 out of 52) (p*=*0.63). In the sub-analysis, in natural cycle group, 10 out of 14 patients with RIF and poor endometrium (group 1), had objectively more regular and thicker endometrium compared to their previous HRT cycles, but only 8 patients had at least 7 mm endometrial thickness assessed by ultrasound. 

However, modified natural cycle showed a 35.7% pregnancy rate on patients with RIF and poor endometrium, in whom pregnancy was never seen, previously on HRT prepared endometrium. One case got pregnant on 6mm regular endometrium. There were no differences in abortion rates between two groups (p=0.6). Between all patients in these three groups (108 patients) there were 2 twin pregnancies. There were 3.8% and 2.3% blighted ovum in group 1 and group 2 respectively. The difference was not statistically significant (p*=*0.10).

**Table I:** Clinical characteristics of patients in groups on *HRT* and *Natural* cycle

**Variables**	**Natural cycle (n=56)**	**HRT (n=52)**	**p-value**
Age*	31.18 ± 5.2	31.1 ± 2.8	0.922
Duration of infertility *	8 ± 2.4	8 ± 2.4	1
No of previous attempt *	3 ± 0.9	3 ± 1	1
No of transferred embryo *	2.8 ± 0.2	2.6 ± 0.4	0.079
Clinical pregnancy**	23 (41.07)	19 (36.53)	0.63
Abortion rate**	1 (1.7)	2 (3.8)	0.60
Multiple pregnancy**	1 (2.3)	1 (1.92)	0.10

p<0.05 considered as significant

HRT: hormone replacement therapy

* Data presented as mean±SD.

** Data presented as n (%)

## Discussion

Endometrial integrity is essential in successful implantation and establishment of pregnancies in both natural and HRT cycles. The type of endometrial preparation may affect endometrial receptivity and further implantation success. To optimize implantation, several methods for endometrium preparation have been proposed. There is still a lack of evidence to show any benefit, or to identify which method of endometrium preparation in FET is more effective than another (14). 

High estradiol concentrations may have an influence on endometrial receptivity. This is the first study which compares pregnancy rates in natural cycle and HRT cycle in patients with repeated IVF failure in FET cycles. Shapiro et* al*, showed impaired endometrial receptivity after hormonal stimulation, comparing clinical pregnancy rates after frozen-thawed and fresh ET (84.0% in the cryopreservation group and 54.7% in the fresh group) (15, 16). So it can be suggested to transfer embryo in non-stimulated endometrium for better embryo-endometrium synchronization. Currently, there are some debates as to whether HRT offers any benefit in comparison with ETs performed during natural cycles (17, 18). 

Queena *et al* showed similar results with programmed and natural cycle in patients with frozen-thawed embryos (19). However, Xiao* et al *showed that natural cycle had a higher trend of clinical pregnancy rate using natural cycle for FET in their study. Also *Chang et al* showed that using natural cycles with or without hCG had better outcomes compared with hormonally manipulated cycles (18, 20). Chai *et al* analyzed expression of estrogen receptors of endometrium in stimulated cycles compared with natural cycles (21). It has been shown that expression of estrogen receptor α (ERα) but not ERβ or progesterone receptor (PR) transcript, significantly reduced in stimulated cycles compared with natural cycles. 

The endometrium of stimulated cycles was shown lower expression of PR protein in glands, but a higher expression in stroma (21). Our study was the first preliminary study, about the possible beneficial effects of natural cycle endometrium for transferring frozen-thawed embryos with a higher clinical pregnancy rate in patients with RIF. Endometrial thickness predicts IVF outcome. It has been shown that pregnancy rates increased significantly as endometrial thickness increased, independent of the number and quality of embryos transferred (22). Thinner endometrium reflects a diminished endometrial responsiveness to ovarian hormones and poor receptivity of the endometrium, leading to a low clinical pregnancy rate. It has been proposed different thresholds of endometrial thickness for successful implantation, but it is suggested that implantation establishes when the thickness of the pre-ovulatory endometrium is <6 mm (23). This is the first study which showed the possible effect of using natural cycle on endometrial receptivity in patients with RIF and thin endometrium.

The limitation of this study was the number of patients with thin endometrial thickness (≤7 mm), the number of subjects in the study population group 1 was too small to make a definitive statement. However, the results showed a 35.7% pregnancy rate in patients with RIF and thin endometrium. Interestingly, there was even one pregnancy with 6 mm endometrium. Although these preliminary results are encouraging, further investigation is needed to approve our results.

We preferred to make use of the modified natural cycle with the administration of HCG for optimizing the timing of FET. Moreover, the endometrium expresses HCG-LH receptors throughout the cycle and the application of HCG significantly increases the intrauterine vascular endothelial growth factor level and cascade of events essential for proper implantation and placentation (24). More than two embryos were transferred in some cases in this study according to the guideline no 182, 2006, recommended in exceptional cases with poor prognoses and RIF cycles (III-C) (25). Modified natural cycle was used by adding luteal phase support similar to regimen in other groups. However, Lee *et al* showed that the pregnancy outcomes on natural cycles were similar with or without luteal phase support (26). 

## Conclusion

In conclusion, the natural cycle had a higher trend of clinical pregnancy rate in comparison with the HRT cycle in patients with RIF and poor endometrium. 
